# Effects of cell adhesion motif, fiber stiffness, and cyclic strain on tenocyte gene expression in a tendon mimetic fiber composite hydrogel

**DOI:** 10.1016/j.bbrc.2018.03.203

**Published:** 2018-05-15

**Authors:** Dharmesh Patel, Sadhana Sharma, Hazel R.C. Screen, Stephanie J. Bryant

**Affiliations:** a School of Engineering and Materials Science, Queen Mary University of London, London, E1 4NS, UK; b Department of Chemical and Biological Engineering, University of Colorado Boulder, Boulder, CO 80309, USA; c Material Science and Engineering Program, University of Colorado-Boulder, Boulder, CO 80309, USA; d BioFrontiers Institute, University of Colorado-Boulder, Boulder, CO 80309, USA

**Keywords:** Tenocyte, Biomimetic hydrogel, Cell adhesion peptide, Stiffness, Mechanotransduction, Cyclic tensile strain

## Abstract

We recently developed a fiber composite consisting of tenocytes seeded onto discontinuous fibers embedded within a hydrogel, designed to mimic physiological tendon micromechanics of tension and shear. This study examined if cell adhesion peptide (DGEA or YRGDS), fiber modulus (50 or 1300 kPa) and/or cyclic strain (5% strain, 1 Hz) influenced bovine tenocyte gene expression. Ten genes were analyzed and none were sensitive to peptide or fiber modulus in the absence of cyclic tensile strain. Genes associated with tendon (*SCX* and *TNMD*), collagens (*COL1A1*, *COL3A1*, *COL11A1*), and matrix remodelling (*MMP1*, *MMP2*, and *TIMP3*) were insensitive to cyclic strain. Contrarily, cyclic strain up-regulated *IL6* by 30-fold and *MMP3* by 10-fold in soft YRGDS fibers. *IL6* expression in soft YRGDS fibers was 5.7 and 3.3-fold greater than in soft DGEA fibers and stiff RGD fibers, respectively, under cyclic strain. Our findings suggest that changes in the surrounding matrix can influence catabolic genes in tenocytes when cultured in a complex strain environment mimicking that of tendon, while having minimal effects on tendon and homeostatic genes.

## Highlights

• A fiber composite material exhibits multimodal shear and tension micromechanics.
• Gene expression of tenocytes is insensitive to fiber stiffness or YRGDS vs DGEA.
• Tendon marker and collagen genes are insensitive to fiber environment under strain.
• *MMP3* and *IL6* genes are sensitive to fiber stiffness and peptide under cyclic strain.
• Healthy tenocytes rapidly respond to their environment by a catabolic response.

## 1 Introduction

Tendons are multiscale composite materials that consist of collagen fibers, which bundle together to create fascicles and ultimately tendon tissue, all aligned in the direction of loading for efficient load transfer between muscle and bone. Tissue extension is achieved by extension and sliding throughout the hierarchy, particularly between collagen fibers and fascicles; the two structural levels where tenocytes reside. As a consequence, tenocytes sitting along collagen fibers within a fascicle experience tension and shear as these units stretch and slide under mechanical loads [[1], [2], [3]]. Overall, this local mechanical environment creates highly complex, anisotropic strains around tenocytes.

Tenocytes regulate their behavior in response to the local environment. The degree and pattern of mechanical loading to which the tissue and cells are exposed impacts tendon development, homeostasis and degeneration [4]. For example, in rat tendons low to moderate fatigue loading enhanced expression of anabolic genes (i.e., collagens) while high fatigue loading enhanced catabolic genes [5]. Fatigue loading of isolated fascicles increased expression of pro-inflammatory genes [6]. Cyclic tensile strain applied to tenocytes led to increased collagen gene expression [7], increased collagen production [8], and decreased matrix metalloproteinase (MMP) gene expression [7]. However, when shear strain was applied to 2D cultures of isolated tenocytes, catabolic genes for MMPs were elevated, concomitant with decreased expression of tissue inhibitors of metalloproteinases (TIMPs) genes [9].

Relating these findings to tenocyte mechanobiology is challenging because the exact nature of the biochemical and local mechanical cues surrounding tenocytes in tissue explants is not well-characterized. While 2D and 3D cultures of tenocytes offer control over the local cues, they do not capture the complex tension and shear micromechanics of tendon. To address this shortcoming, we recently developed a novel fiber composite hydrogel that under cyclic tensile strains recapitulates the complex micromechanical environment surrounding tenocytes with simultaneous shear strain, created from fiber sliding, and tensile strain, created from fiber stretch [10].

The goal of this study was to employ this new fiber composite hydrogel [10] to investigate whether the cell adhesion peptide and fiber stiffness affect tenocyte gene expression when combined with cyclic tensile strains. Fibers of two stiffness were fabricated with either cell adhesion peptide, DGEA (Asp-Gly-Glu-Ala) or YRGDS (Tyr-Arg-Gly-Asp-Ser) to mimic cellular interactions to collagen type I [11] or fibronectin [12], respectively. Both proteins are found in the pericellular matrix surrounding tenocytes in their native environment [13]. Primary bovine tenocytes seeded within the fiber composite hydrogels were evaluated by gene expression for tendon markers, anabolic genes, and catabolic genes.

## 2 Materials and methods

### 2.1 Tenocyte source

Primary tenocytes were isolated from bovine common digital extensor tendons from three donors (1.5–2 years old) (Arapahoe Meat Co, USA) and kept separate (n = 3). Tendon digestion was achieved by 1 U/ml dispase (STEMCELL Technologies, USA) and 2 mg/ml collagenase type II (Worthington Biochemical Corporation, USA) for 48 h. Isolated tenocytes were suspended in culture medium without fetal bovine serum (FBS) and used immediately. Culture medium consisted of DMEM with low glucose and pyruvate (Life Technologies, USA), 10 mM HEPES buffer (Sigma Aldrich, USA), 4 nM L-glutamine (Sigma Aldrich, USA), 0.1 M non-essential amino acids (Sigma Aldrich, USA), 50 U/ml penicillin and 50 μg/mL streptomycin (Sigma Aldrich, USA).

### 2.2 Syntheses of monomers

**Fig. 1:**
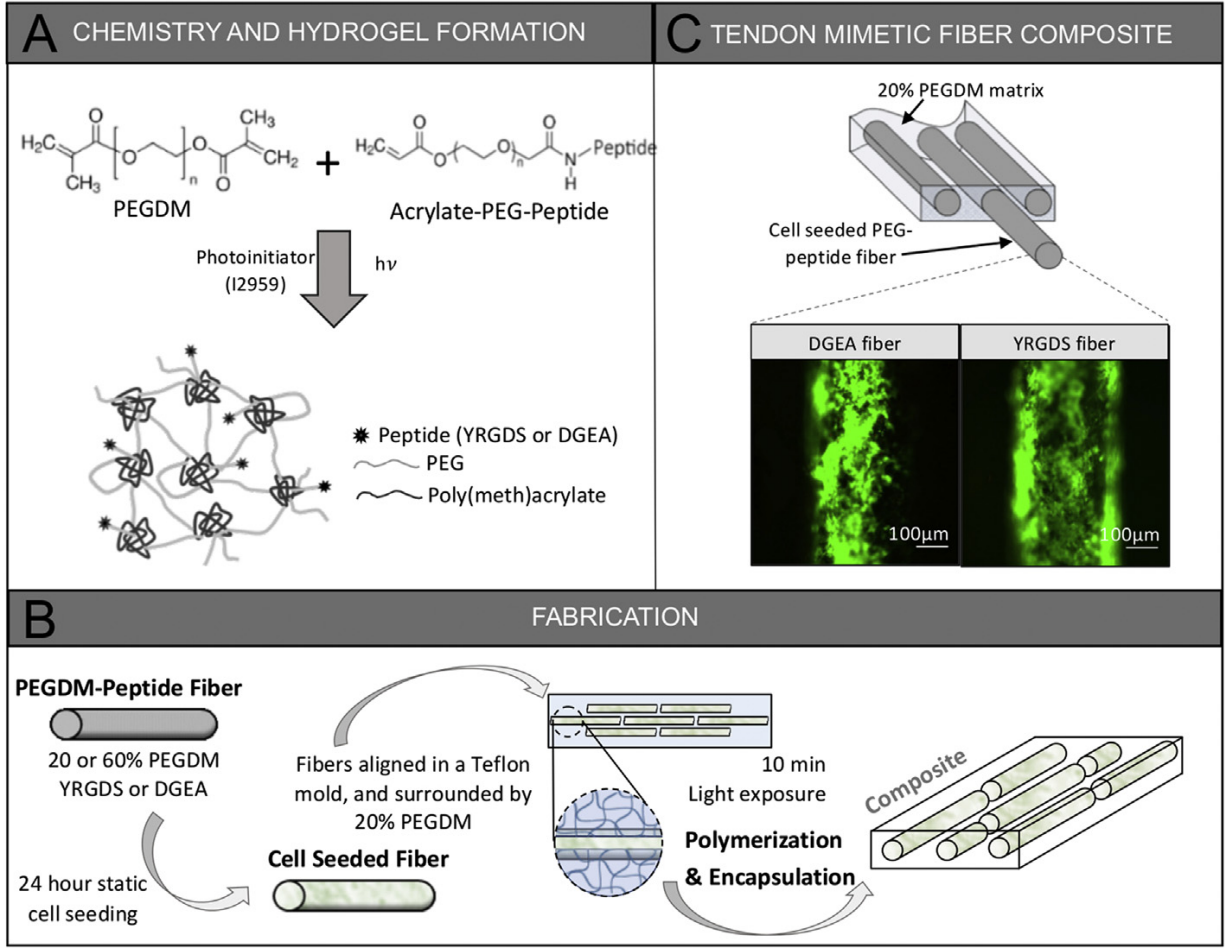
Tendon mimetic hydrogel chemistry (A) and fabrication process (B). Viable tenocytes (green) are shown adhered to PEG-peptide fibers embedded within a PEG hydrogel matrix. (For interpretation of the references to colour in this figure legend, the reader is referred to the Web version of this article.)

Poly(ethylene glycol) dimethacrylate (PEGDM) (Fig. 1A) was synthesized by reacting PEG (3000 MW, Merck Schuchardt, Germany) with 5 molar excess methacrylic anhydride (Sigma Aldrich, USA) with trace hydroquinone (Sigma Aldrich, USA) for 10 min at 400 W [14]. PEGDM was purified by precipitation in diethyl ether. The degree of methacrylate substitution was 93% by ^1^H-NMR spectroscopy.

Acrylate-PEG-peptide (Fig. 1A) monomer containing peptide was prepared by reacting acrylate-PEG-succinimidyl valerate (3400 Da; Laysan Bio, Inc.) with 10% molar excess peptide (YRGDS or DGEA, GenScript, USA) in 50 mM sodium bicarbonate buffer (pH 8.4) at room temperature for two hours. The resulting conjugate was purified by dialysis against deionized water, and lyophilized. The degree of peptide functionalization was determined to be >93% by Fluoraldehyde™ o-Phthalaldehyde (Pierce, USA).

### 2.3 Fiber composite manufacture

Fiber stiffness was controlled by PEGDM concentration prior to polymerization. Fibers were made from 20% or 60% (w/v) PEGDM and 5 mM acrylate-PEG-peptide in phosphate buffered saline (PBS) (pH 7.4, Sigma Aldrich, USA) with 0.05% w/v Irgacure 2959 (BASF, USA). This solution was polymerized in a Teflon mold (0.3 mm diameter and 4 mm length) under 352 nm light (∼5 mW/cm^2^) for 10 min. The fibers were sterilized in 70% ethanol overnight, under short wavelength UV light in a sterile hood for one hour, and then rinsed 3× with sterile PBS. Sterile fibers (∼150) were placed in non-tissue culture treated 48-well plates, incubated with tenocyte culture medium without FBS for 30 min, and then seeded with 3.5 million tenocytes per well in tenocyte culture medium without FBS for 1.5 h to support cell attachment. Cells were then supplemented with 5% serum, cultured for another 1.5 h, and finally cell seeded fibers were transferred to a new well and cultured in complete tenocyte medium with 10% FBS overnight.

Fiber composites were fabricated by aligning ∼7 tenocyte-seeded fibers approximately parallel to each other, but staggered (Fig. 1B) in a sterile rectangular Teflon mold (25 mm × 2.5 mm x 1 mm), spanning the entire length. A sterile filtered precursor solution of 20% (w/w) PEGDM with 0.05% (w/v) Irgacure 2959 in tenocyte culture medium without FBS was slowly injected around the fibers to minimize fiber movement. The solution was then exposed to 352 nm light (∼5 mW/cm^2^, 10 min). Manufactured fiber composites were maintained in complete tenocyte culture medium incubated at 37 °C with 5% CO_2_ for 24 h.

### 2.4 Tenocyte viability

Tenocyte viability was measured in the fiber composites immediately after encapsulation by incubating with calcein AM (4 μM) and ethidium homodimer (4 μM). Tenocytes were imaged at two locations per fiber (*n* = 3 samples) with an epifluorescent microscope (DMI 4000B, Leica) at 10× magnification.

### 2.5 Mechanical properties of fibers and fiber composites

Hydrated samples were placed into a Hounsfield tensile test machine with a 5N load cell. Samples were tested (n = 9-13 per sample type) to failure at a strain rate of 15%/min.

### 2.6 Tensile strain measurement

Fiber strain within tenocyte-seeded fiber composites was assessed using a custom uniaxial strain rig with brightfield microscopy [15]. Tenocytes at passage four from one donor and fibers with YRGDS were used to create composites with the 20% (n = 12) and 60% (n = 6) PEGDM fibers. Fibers within composites were imaged before and after applying a 5% tensile strain at 15%/min. Images were captured and 2–6 fibers analyzed per sample using NIH ImageJ software. Local fiber tensile strains were calculated by dividing fiber extension by original fiber length.

### 2.7 Cyclic tensile strain application

A separate set of fiber composites were manufactured (as described above) using freshly isolated tenocytes from three different donors. Composites were placed into individual wells in 6-well plates (free-swelling) or a sterile custom bioreactor (loaded) [35] in an incubator at 37 °C and 5% CO_2_, and left to stabilize for 24 h. The loaded sample was subjected to a 5% amplitude strain applied in a sinusoidal waveform at 1 Hz continuously for 24 h. For each substrate stiffness and peptide motif, there were two loaded samples and two free swelling samples per biological replicate.

### 2.8 RNA extraction and RT-qPCR

RNA was extracted using a miRNeasy Micro Kit (QIAgen, USA) from freshly isolated tenocytes of each donor (referred to as donor tenocytes) and from tenocyte-seeded fiber composites at experiment's end. Samples were snapped frozen in liquid nitrogen and homogenized (TissueLyser II). Technical replicates for each experimental condition and donor were pooled to obtain enough RNA. RNA (180 ng) was reverse transcribed to cDNA (High-Capacity cDNA Reverse Transcription (RT) Kit, Applied Biosystems). Quantitative real-time polymerase chain reaction (qPCR) was performed using Fast SYBR Green Master Mix (Applied Biosystems) and custom primers (Supplemental Table 1) (Life Technologies, USA) on a 7500 Fast Real-Time PCR Machine (Applied Biosystems). Gene expression for each gene of interest (GOI) was analyzed following the Pfaffl method using primer efficiency (*E*) [16] relative to the reference gene (REF), ribosomal protein L30, *RPL30*. Gene expression data are reported as relative expression (RE) by

$$RE=E_{REF}^{C_{T\left(REF\right)}}/E_{GOI}^{C_{T\left(GOI\right)}}$$

or normalized expression (NE) by

$$NE=E_{GOI}^{\Delta C_{T\left(GOI\right)}\left(calibrator-sample\right)}/E_{REF}^{\Delta C_{T\left(REF\right)}\left(calibrator-sample\right)}$$

where *C*_*T*_ is the cycle threshold point, the calibrator is the freshly isolated donor tenocytes and the experimental sample.

### 2.9 Statistical analysis

Data are reported as the mean with standard deviation denoted parenthetically in the text or as error bars. All statistical tests were performed using Real Statistics add-in for Excel. Data were tested for normality using the Shapiro-Wilk test and analyzed for statistical significance. The mechanical properties were compared using a *t*-test or a one-way ANOVA (α = 0.05). Gene expression was analyzed using a three-way ANOVA (α = 0.05) with peptide type (DGEA vs YRGDS), fiber stiffness, and loading (free swelling vs. cyclic strained) as factors. If the three-way interaction was statistically significant as determined by *p* < 0.05, follow up tests were performed by a simple two-way interactions and simple main effects. Post-hoc analysis was performed using Tukey's HSD with α = 0.05. *P*-values of pairwise comparisons less than or equal to 0.1 are provided to indicate the level of significance [17].

## 3 Results

### 3.1 Fiber composite manufacture

The tendon mimetic hydrogel was fabricated from PEG dimethacrylate and acrylated-PEG-peptide monomers (Fig. 1A and B). The fiber composite hydrogel consisted of soft or stiff tenocyte-seeded PEG fiber-like structures embedded within a soft PEG matrix. The fibers were discontinuous and placed in an aligned and staggered configuration (Fig. 1B). Peptide motifs (DGEA or YRGDS) were tethered into the fibers only to selectively promote cell attachment to the fibers. Tenocytes remained viable during the fabrication process and were localized to the fiber-like structures (Fig. 1C).

### 3.2 Mechanical properties of the fiber composite

**Fig. 2:**
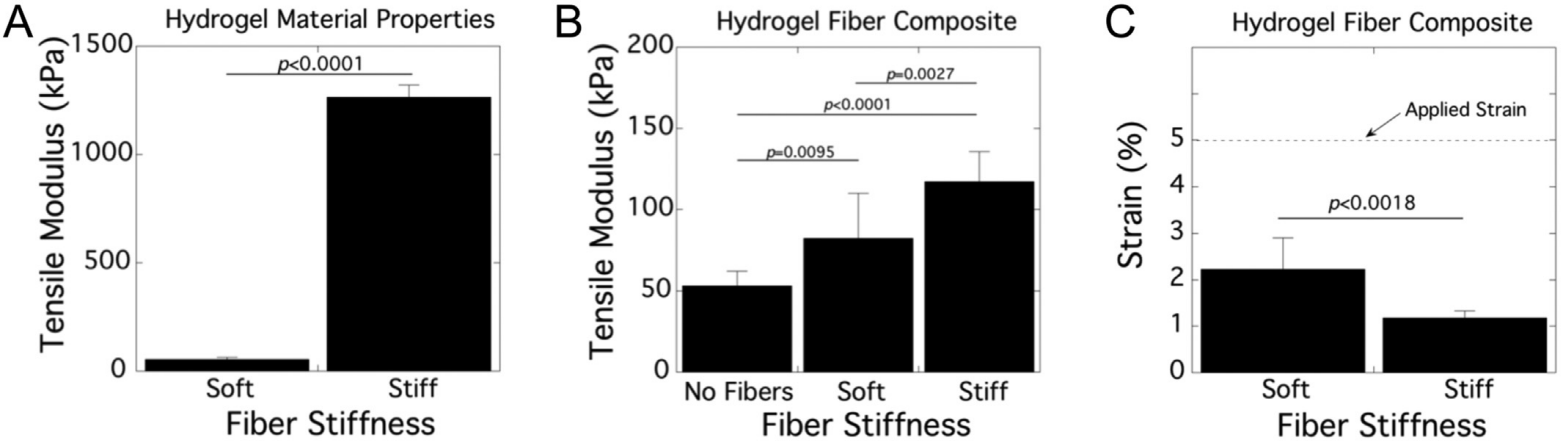
Tensile modulus of soft and stiff fibers (A) and hydrogel fiber composites containing none, soft or stiff fibers (B). Local fiber strain under a 5% applied tensile strain (C). Data are mean with standard deviation as error bars.

The mechanical properties of the tendon mimetic hydrogels and their constituent parts were characterized (Fig. 2). The 20% and 60% PEGDM, used to make the soft and stiff fibers, respectively, had tensile moduli of 53(9) kPa and 1300(56) kPa, respectively (Fig. 2A). The soft hydrogel matrix was prepared from the same formulation as the soft fibers. The stiffness of the matrix was sufficiently high to enable clamping for mechanical testing and stretching in the bioreactor, but not too high to restrict nutrient diffusion to the tenocytes. The tensile modulus of cell-laden fiber composites was compared to that of the hydrogel material without fibers (Fig. 2B). The modulus increased (*p* = 0.0095) by 54% with soft fibers and increased (*p* < 0.0001) by 120% with stiff fibers. Fiber composite micromechanics were determined by measuring fiber strain after applying 5% strain to the fiber composite. For both soft and stiff fibers, fiber strain was less than the overall strain applied, but was dependent on the fiber stiffness (Fig. 2C).

### 3.3 Gene expression of donor tenocytes

**Fig. 3:**
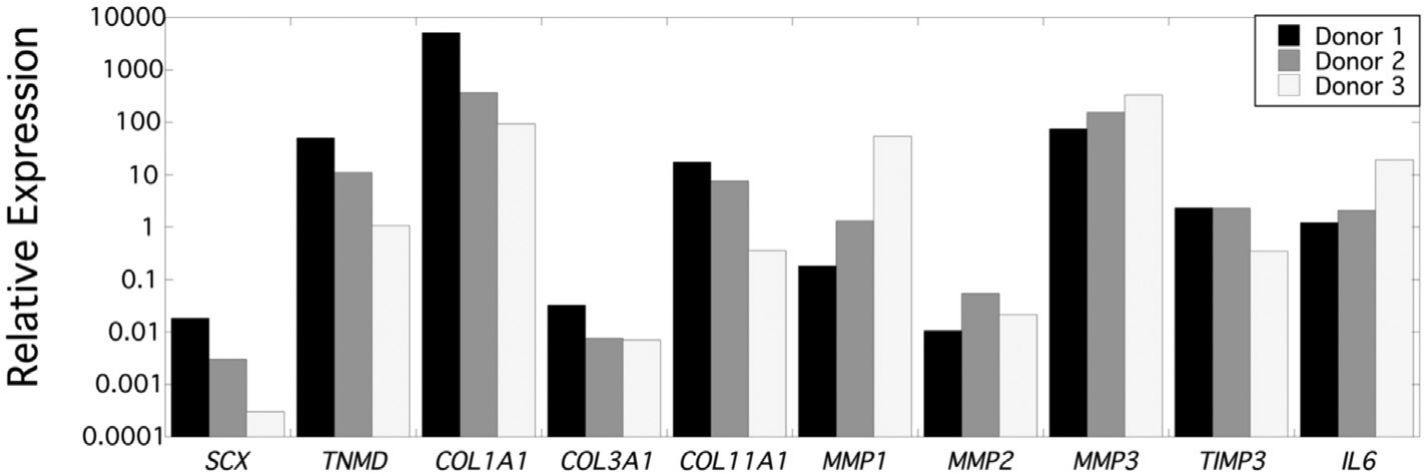
Relative gene expression in tenocytes from each donor directly after harvest, and prior to seeding within the fiber composites.

Gene expression of tenocytes isolated from each donor was evaluated directly after harvest, and prior to seeding within the fiber composites (Fig. 3). Genes associated with tendon markers, anabolism and catabolism were assessed. The relative expression (*RE*) for all genes differed with donor, but trends were similar. *RE* for the tendon marker *TNMD* was higher than *SCX*. Of the collagen genes examined, *COL1A1* expression was highest by ∼2–3 orders of magnitude when compared to *COL11A1* and by ∼6 orders of magnitude when compared to *COL3A1*. Of the MMPs examined, *MMP3* was the highest followed by *MMP1* and then *MMP2*. *TIMP3* levels were lower than *MMP3*, but higher than *MMP2*. Donor 1 had the highest expression of tendon markers and collagen genes, which coincided with the lowest levels of *MMP1, MMP3*, and *IL6*. Donor 3 exhibited an opposite trend, having the lowest expression of tendon markers and collagen genes, but highest levels of *MMP1*, *MMP3*, *TIMP3,* and *IL6*.

### 3.4 Effect of peptide motif, fiber stiffness, and loading on tenocyte gene expression for tendon markers and collagen genes

**Fig. 4:**
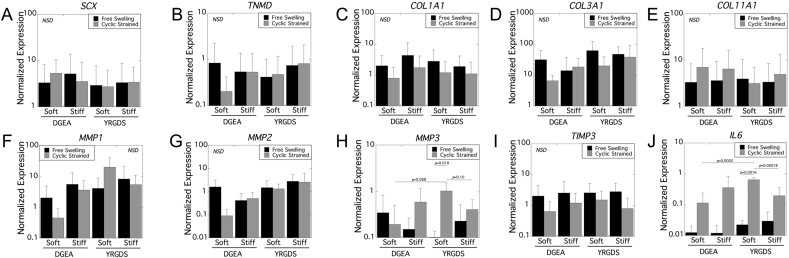
Normalized gene expression as a function of peptide motif, fiber stiffness, and loading after 24 h of culture. Data are presented as mean with standard deviation as error bars (n = 3 donors).

Tenocyte gene expression for tendon markers, *SCX* or *TNMD* (Fig. 4A and B), and collagen genes, *COL1A1*, *COL3A1*, and *COL11A1* (Fig. 4C–E), was investigated as a function of peptide motif, fiber stiffness, and loading. GOI expression was normalized to the relative expression level in the pre-seeded donor tenocytes. Three-way ANOVA results with peptide motif (DGEA or YRGDS), fiber stiffness, and loading (free swelling or cyclic strain) as factors revealed no significant three-way or two-way interactions for the genes investigated. Moreover, there were no significant main effects for the genes investigated.

### 3.5 Effect of peptide motif, fiber stiffness, and loading on tenocyte gene expression for catabolic-related genes

Tenocyte gene expression for matrix degrading enzymes, *MMP1, MMP2* and *MMP3* (Fig. 4F–H), a regulator of matrix degrading enzymes, *TIMP3* (Fig. 4I), and a pro-inflammatory cytokine, *IL6* (Fig. 4J), was investigated as a function of peptide motif, fiber stiffness, and loading. GOI expression was normalized to the relative expression level in the pre-seeded donor tenocytes. Three-way ANOVA results with fiber stiffness, loading, and peptide motif as factors revealed no significant three-way or two-way interactions and no significant main effects for *MMP1*, *MMP2*, and *TIMP3*.

There was a significant three-way interaction for *MMP3* (*p* = 0.04) expression. Follow-up two-way analyses were performed and revealed several findings. For YRGDS, loading was a significant factor (*p* = 0.017), but not stiffness. Cyclic strain up-regulated (*p* = 0.018) *MMP3* expression for the soft YRGDS fibers. Mean *MMP3* expression was higher (*p* = 0.10), but not statistically significant, for the soft YRGDS fibers compared to the stiff YRGDS fibers under loading. For DGEA, loading and stiffness were not significant factors. Under free swelling conditions or cyclic strain, peptide and stiffness were not significant factors. For soft fibers, there was a significant two-way interaction (*p* = 0.04) between peptide and loading, which revealed a higher (*p* = 0.068), but not statistically significant, mean *MMP3* expression for the YRGDS soft fibers compared to the DGEA soft fibers. For stiff fibers, peptide motif and loading were not significant factors.

There was also a significant three-way interaction for *IL6* (*p* = 0.04) expression. Follow-up two-way analyses were performed, which revealed several findings. For YRGDS, loading (*p* = 0.00015) and stiffness (*p* = 0.0053) were significant factors and there was a significant two-way interaction (*p* = 0.0045). Specifically, cyclic strain up-regulated (*p* = 0.0016) *IL6* expression for the soft fibers. *IL6* expression was significantly higher (*p* = 0.00018) for the soft YRGDS fibers compared to the stiff YRGDS fibers under dynamic loading. For DGEA, loading and stiffness were not significant factors. Under free swelling conditions or cyclic strain conditions, peptide and stiffness were not significant factors. For soft fibers, peptide motif (*p* = 0.00071) and loading (*p* = 0.0001) were significant factors and there was a significant two-way interaction (*p* = 0.0009). Specifically, *IL6* expression was significantly higher (*p* = 0.0002) for the YRGDS soft fibers compared to the DGEA soft fibers under cyclic strain. For stiff fibers, peptide motif and loading were not significant factors.

## 4 Discussion

This study employed our recently developed tendon mimetic hydrogel, which similar to tendon, is a fiber composite material that exhibits multimodal shear and tension micromechanics under strain. Results from this study demonstrate that tenocyte genes of tendon markers, collagens, matrix remodeling, and inflammatory genes, were not sensitive to peptide motif or fiber stiffness in the absence of loading. Tendon markers and collagens were not sensitive to loading. Contrarily, *MMP3* and *IL6* were elevated under loading with YRGDS soft fibers showing the most significant effect. These results suggest that catabolic and inflammatory genes may be more sensitive than anabolic genes to differences in the local environment under cyclic tensile strain.

In the tendon mimetic hydrogel, tenocytes adhere to fibers, which are encapsulated into a hydrogel matrix. During the encapsulation process, monomers diffuse into the fibers creating an interpenetrating network, which effectively increases hydrogel crosslink density at the boundary. This interpenetrating network increases the mechanical properties leading to the higher tensile modulus for the soft fiber composite compared to no fibers despite being from the same material. With increasing fiber stiffness, tensile modulus was even higher. When the tendon mimetic hydrogel was subjected to tensile strains, fibers stretched, but not to the same degree as the overall strain applied to the hydrogel. The stiffer the fiber, the less the fibers stretched. Fiber strain ranged from 1.2% to 2.2%, which is within the range reported for healthy tendons under a 5% applied strain [10]. Therefore, the tendon mimetic hydrogel under conditions studied herein represent healthy tendon micromechanics.

Gene expression for tendon markers, scleraxis and tenomodulin, were not affected by peptide motif, fiber stiffness, or loading. Scleraxis is an early stage marker of tendon development [18]. Tenomodulin is a marker of maturation and is regulated by scleraxis [19]. Studies have reported early increases and substrate-dependent effects in *SCX* expression in stem cells during tenogenesis [20]. Similarly, *TNMD* expression is upregulated during tenogenic differentiation of stem cells [21]. On the contrary, mature tenocytes were used herein and their expression levels remained similar to the pre-seeded tenocytes, suggesting that the mature tenocytes maintained their tenogenic phenotype within the tendon mimetic hydrogel regardless of the environment, which is consistent with other studies [22].

Tenocyte gene expression for collagen genes, *COL1A1*, *COL3A1*, and *COL11A1* were not affected by peptide motif, fiber stiffness, or loading. Collagen type I is a fibril-forming collagen and the main collagen of tendons. Collagen type III is a fibril-forming collagen that is upregulated during tendon healing [23]. Collagen type XI is directly associated with collagen types I and III and is involved in fibrillogenesis in developing tendons [24]. Additionally, MMP1 and MMP2, which are functionally related to collagen degradation [48], and TIMP3, which regulates many MMPs, were not affected by these factors. Our studies suggest that under normal physiological strain levels, genes for collagens and their associated MMPs and MMP regulators are not sensitive to tenocyte environment under the conditions studied herein.

Of the catabolic and inflammatory genes investigated, *MMP3* and *IL6* were up-regulated most significantly by loading in soft YRGDS fibers. MMP3 is known for activating other MMPs, which are involved in matrix degradation [25], and for degrading proteoglycans and non-fibrillar collagens. *IL6* expression is induced during inflammation [26] and its upregulation has been reported in ruptured and painful tendons in humans [27]. YRGDS is a primary integrin-binding domain in fibronectin [28], which is at high levels immediately following injury [23] and is prevalent in the ECM of ruptured tendon [29]. The large presence of YRGDS in the tendon mimetic hydrogel may simulate an initial injury. Under cyclic tensile strain, fiber strains were within reported physiological strains of healthy tendon. This observation suggests that fiber stiffness may be a contributing factor to elevated tenocyte catabolic response when combined with loading, which is consistent with microdamage, which occurs after injury and weakens tendon [30]. Given these findings, we postulate that *MMP3* and *IL6* may be early responders to changes in the tenocyte environment where YRGDS and soft fibers may mimic the initial injury response.

There are several limitations of this study. This study was limited to gene expression and a short culture time. The latter was necessary to maintain control over the chemical and mechanical cues in the microenvironment and to minimize contributions from tenocyte deposited matrix. Long-term studies are needed to assess protein secretion to determine the potential autocrine and paracrine effects, especially when pro-inflammatory cytokines are up-regulated.

In conclusion, a tendon mimetic hydrogel that affords control over the microenvironment enabled investigation into the role of biochemical and mechanical cues in regulating tenocyte gene expression. This study demonstrates that gene expression of bovine tenocytes was largely insensitive to their environment in the absence of loading. Under cyclic strain, a subset of catabolic genes was sensitive to the peptide motif and fiber stiffness. Our findings suggest that healthy tenocytes can sense their local strain environment and depending on the peptide motif and stiffness, initiate a catabolic response. Findings from this study suggest that changes to the composition and/or structure of the tendon could initiate a tenocyte-mediated catabolic response, thus warranting further research.

## Appendix A Supplementary data

Patel Supplemental Patel Supplemental
