# Supplementary material for: Effects of cell adhesion motif, fiber stiffness, and cyclic strain on tenocyte gene expression in a tendon mimetic fiber composite hydrogel
Source: Biochem Biophys Res Commun. 2018 May 15;499(3):642–7. doi: 10.1016/j.bbrc.2018.03.203 (PMC5916510; doi:10.1016/j.bbrc.2018.03.203)
Supplement: Patel Supplemental [file mmc1.docx]

| **Supplemental Table 1. Primers used for RT-qPCR** | | | |
| --- | --- | --- | --- |
| **Category** | **Name** | **Gene** | **Primer Sequence** |
| Tendon Markers | Scleraxis | *SCX* | F: 5’-GAGAAAGTTGGTGAGTGTTGC-3’  R: 5’-GGGTGGTTTGGAGGTGG-3’ |
|  | Tenomodulin | *TNMD* | F: 5’-TCTGGCGTGACGGGTCTT-3’  R: 5’-AAAAAAGGCATTGAACAAAACGA-3’ |
| Anabolism | Collagen Type I A1 | *COL1A1* | F: 5’-GCCTGGTCAGAGAGGAGAAAGA-3’  R: 5’-CCTTGTTTGCCGGGTTCAC-3’ |
|  | Collagen Type III A1 | *COL3A1* | F: 5’-CTGACATTTAGACATGATGAG-3’  R: 5’-ACTGACCGAGATGGGAGCAT -3’ |
|  | Collagen Type XI A1 | *COL11A1* | F: 5’-GTCTACCAGGTGACAAGGGTC-3’  R: 5’-CGGCTATACCAGGCTGTCC-3’ |
| Catabolism | Matrix Metalloroteinase-1 | *MMP1* | F: 5’-GCTTTCTCAGGACGACATTGATG-3’  R: 5’-CGACTGGCTGAGTGGGATTT-3’ |
|  | Matrix Metalloproteinase-2 | *MMP2* | F: 5’-TACGACCGCGACAAGAAGTAT-3’  R: 5’-TTGTTGCCCAGGAAAGTGAAG-3’ |
|  | Matrix Metalloproteinase-3 | *MMP3* | F: 5’-TCCGCCTTTCTCAGGATGAT-3’  R: 5’-GGCACCACAGGGTCATTAGG-3’ |
|  | Tissue Inhibitor of Metalloproteinase-3 | *TIMP3* | F: 5’-AGTCTCTGTGGCCTTAAGCTTGA-3’  R: 5’-TGGTCCCACCTCTCTACAAAGTTAC-3’ |
|  | Interleukin-6 | *IL6* | F: 5’-CCAGACAAAACCGAAGCTCTCA-3’  R: 5’-CTCATCATTCTTCTCACATATCTCCTTT-3’ |
| Reference | Ribosomal protein L30 | *RPL30* | F: 5’-GGCAGGCGGATTCTTTACC-3’  R: 5’-TAGAGGCTTTCTCTCGGACAGATG-3’ |
|  | | | |
